# Potential for sub-mm long erbium-doped composite silicon waveguide DFB lasers

**DOI:** 10.1038/s41598-020-67722-y

**Published:** 2020-07-02

**Authors:** Zhengrui Tu, Jianhao Zhang, John Rönn, Carlos Alonso-Ramos, Xavier Leroux, Laurent Vivien, Zhipei Sun, Éric Cassan

**Affiliations:** 1 Université Paris-Saclay, CNRS, Centre de Nanosciences et de Nanotechnologies, 91120 Palaiseau, France; 2 Department of Electronics and Nanoengineering, Aalto University, Tietotie 3, 00076 Espoo, Finland; 3 Department of Applied Physics, QTF Centre of Excellence, Aalto University, 00076 Aalto, Finland

**Keywords:** Silicon photonics, Optoelectronic devices and components

## Abstract

Compact silicon integrated lasers are of significant interest for various applications. We present a detailed investigation for realizing sub-mm long on-chip laser structures operating at λ = 1.533 µm on the silicon-on-insulator photonic platform by combining a multi-segment silicon waveguide structure and a recently demonstrated erbium-doped thin film deposition technology. Quarter-wave shifted distributed feedback structures (QWS-DFB) are designed and a detailed calculation of the lasing threshold conditions is quantitatively estimated and discussed. The results indicate that the requirements for efficient lasing can be obtained in various combinations of the designed waveguide DFB structures. Overall, the study proposes a path to the realization of compact (< 500 µm) on-chip lasers operating in the C-band through the hybrid integration of erbium-doped aluminum oxide processed by atomic layer deposition in the silicon photonic platform and operating under optical pumping powers of few mW at 1,470 nm.

## Introduction

Silicon photonics has drawn a great interest in the past decades^1,2^. The maturity of this field is such that its transition to an industrialization stage has already been achieved with the most important applications in the field of telecommunications and datacom^3–7^. Nevertheless, there are still key points to be resolved, including the issue of the light source. Due to silicon’s indirect band gap, one of the biggest challenges in silicon photonics is to realize compact, high efficiency, low power consumption and low cost on-chip lasers and amplifiers^8^. Several methods have been investigated to address this problem. The hybrid integration of III/V based lasers on silicon can lead to high efficiency integrated lasers and is considered as the present dominant approach^9^. This integration scheme is clearly controlled and effective, as the question of light coupling between active III/V zones and silicon waveguides has been solved in previous works^10,11^. It nevertheless requires heterogeneous integration technologies, which are not directly complementary metal oxide semi-conductor (CMOS) compatible with additional fabricating process steps. Any direct monolithic integration of materials deposited at temperatures compatible with a back-end CMOS process (≤ 500 °C) is therefore preferable. In this way, introducing rare-earth doped materials wildly employed in the development of optical fiber communication systems may be of interest^12^.

Recently, several key progresses related to the integration of rare-earth-doped amorphous aluminum oxides in silicon nitride waveguides have been reported^13–22^. These works have enabled very interesting prospects by proposing integrated structures that have led to laser emission in several configurations, the most interesting one being probably that of optically pumped integrated DFB lasers. However, the demonstrated devices have footprint sizes of more than 2 cm^14^ or even much larger^22^. By comparing these dimensions with those required by advanced silicon photonics integration schemes, they appear as significantly larger than the usual dimensions of typical waveguides, micro-resonators, and active components. An effort to miniaturize erbium-doped optical lasers is therefore required. Another limitation not addressed by the previous works is the choice of the pump wavelength. In most of works, the active medium is pumped at 980 nm, which allows one to produce strong population inversion in the active medium due to high absorption cross-section of Er at the corresponding wavelength. However, pumping at 980 nm cannot be realized in Si waveguides due to low transparency of Si at wavelengths shorter than λ = 1.1 µm.

In this context, the objective of this article is to address these two limitations and to propose a possible approach for the realization of erbium-doped lasers on silicon, pumped at ~ 1.48 µm, directly integrated in silicon waveguides, with sub-mm dimensions. To this end, our study is based on the gain material we have recently developed^23^. With a 1,470 nm pump source, up to 52.4 ± 13.8 dB/cm (12.07 cm^−1^) net material gain per unit length at 1533 nm wavelength was demonstrated, which, to the best of our knowledge, is among the highest gain values achieved from erbium-based planar composite waveguides. Given such erbium-doped material properties, a proper optical cavity remains to be investigated while considering the most appropriate integration approach of the active layer within the resonator geometry. Inspired by the multi-segment waveguide structure proposed in Ref.^22^ and by taking advantage of our erbium-doped material platform realized with the atomic layer deposition (ALD) technique, we investigate the feasibility for realizing compact (sub-mm) erbium-doped on-chip lasers based on the silicon on insulator (SOI) platform through the design of quarter-wave shifted (QWS) distributed feedback (DFB) cavities designed in multi-rail silicon waveguides.

## Results

### Waveguide structure and analysis of confinement factor

A preliminary step in the design of active optical cavities that can lead to efficient lasing is the design of the active waveguide in which light propagates. Two key elements driving the design of such waveguides are essentially to minimize the effect of two-photon absorption (TPA) in silicon^24^ and to maximize the overlap of the propagating optical mode and the active material deposited on the silicon waveguide with the ALD technique^23^. These two constraints act in the same direction and lead us to predict our choice towards guiding structures with a high deconfinement of the field outside the silicon core. In order to satisfy the two design requirements, we use the multi-segment waveguide structure proposed in Refs.^16–22^ for silicon nitride waveguides but transpose and apply it to silicon waveguides while taking into account the substantial index contrast increment between the SiN and SOI waveguides.

**Figure 1:**
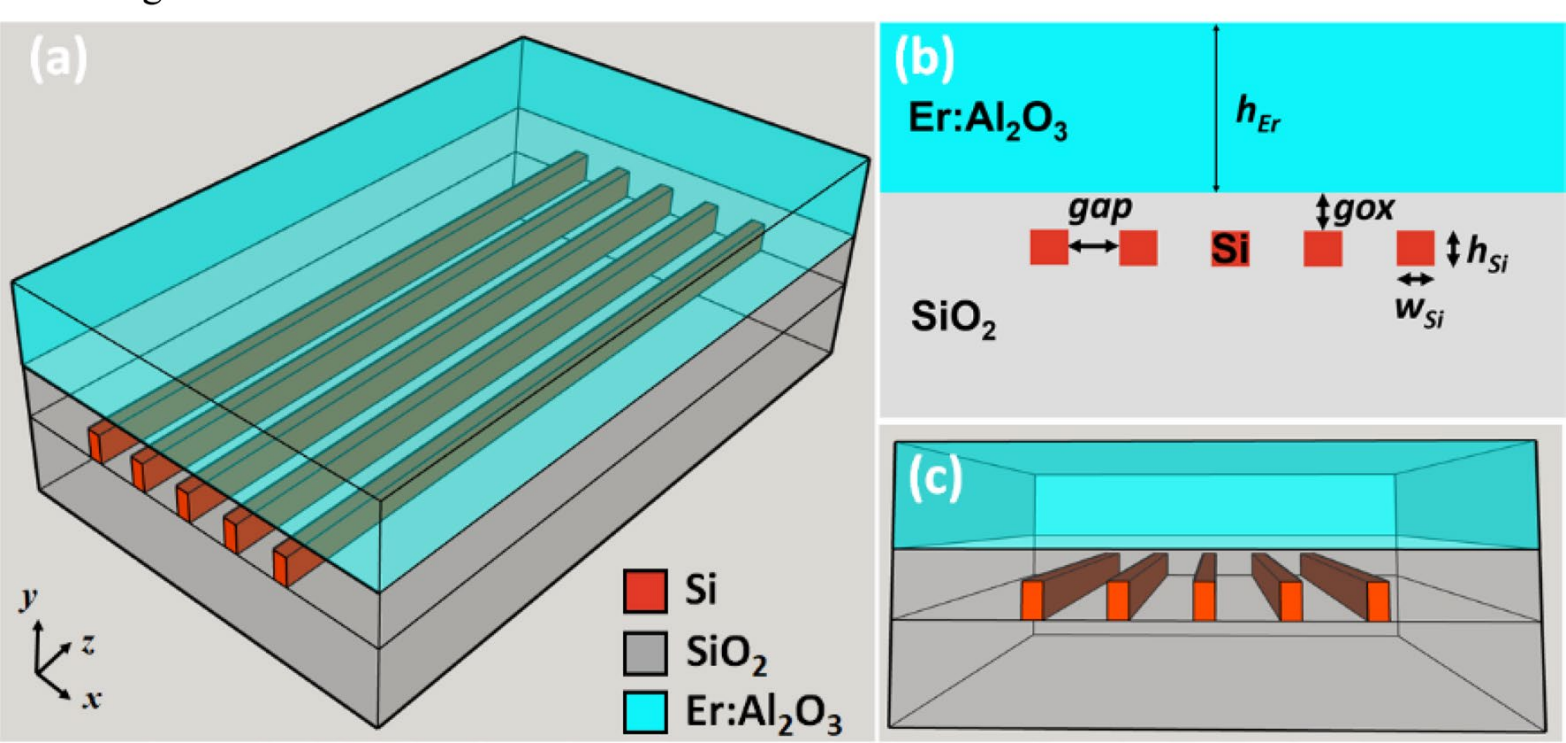
(**a**) 3D schematic diagram of the full waveguide structure. (**b**) Waveguide structure cross section view. (**c**) Front view of the waveguide structure.

The 3D schematic diagram of waveguide is shown in Fig. 1a. Based on a standard 220 nm thick silicon core SOI wafer, etching of five silicon segments can be performed, followed by a SiO_2_-layer deposition upon the silicon-segments. Figure 1b shows the cross-section of the waveguide structure and Fig. 1c shows its front view. This thin SiO_2_ layer on top of the Si-segments can reduce the impact of the high refractive index Si wires on the optical mode distribution and improve the wavelength insensitivity to obtain a higher overlap between the pump and signal modes^22^. Finally, an Er:Al_2_O_3_ thin film can be grown on top the structure with the ALD technique. The main waveguide related parameters are labeled in Fig. 1b. The thickness of the thin SiO_2_ layer between the Si-segments and the Er:Al_2_O_3_ layer is labeled as *gox.* Si-segments have a width of *w*_*Si*_, a thickness of *h*_*Si*_ and an inter-segment distance referred as *gap*. The thickness of the active cladding is labeled as *h*_*Er*_.

As previously stated, a key point in this work is to consider pumping at 1,470 nm light wavelength to coincide with our previous results that showed a net material gain of ~ 52 dB/cm at 1533 nm wavelength^23^. An advantage of the significant proximity between the signal and pump wavelengths (difference of 4.3%) is that the modal distributions of the fields at these two wavelengths are very close, thus improving their spatial overlap and providing very similar single mode waveguide conditions. In order to identify the optimal propagation conditions and to evaluate the modal overlap factor of the modes with the active material, we calculated the mode profiles at these two wavelengths in different configurations using a finite element mode solver (*COMSOL Multiphysics*). The refractive indices of the related waveguide materials are listed in Table 1. To maximize the mode confinement factor of the optical beams with the active layer, *w*_*Si*_ and *h*_*Si*_ were varied from the minimal feature size values compatible with the technological clean room limitations (e.g. 50 nm) up to the maximum values to ensure single-mode operation. Meanwhile the inter-segment distance value (*gap*) was similarly considered to ensure the single mode behavior of the multi-segment waveguide.

**Table 1:** Refractive indices of the materials.

Wavelength	1533 nm	1,470 nm
Material	n	n
Si	3.4771	3.4827
SiO_2_	1.4442	1.4450
Er:Al_2_O_3_	1.6500	1.6501

**Figure 2:**
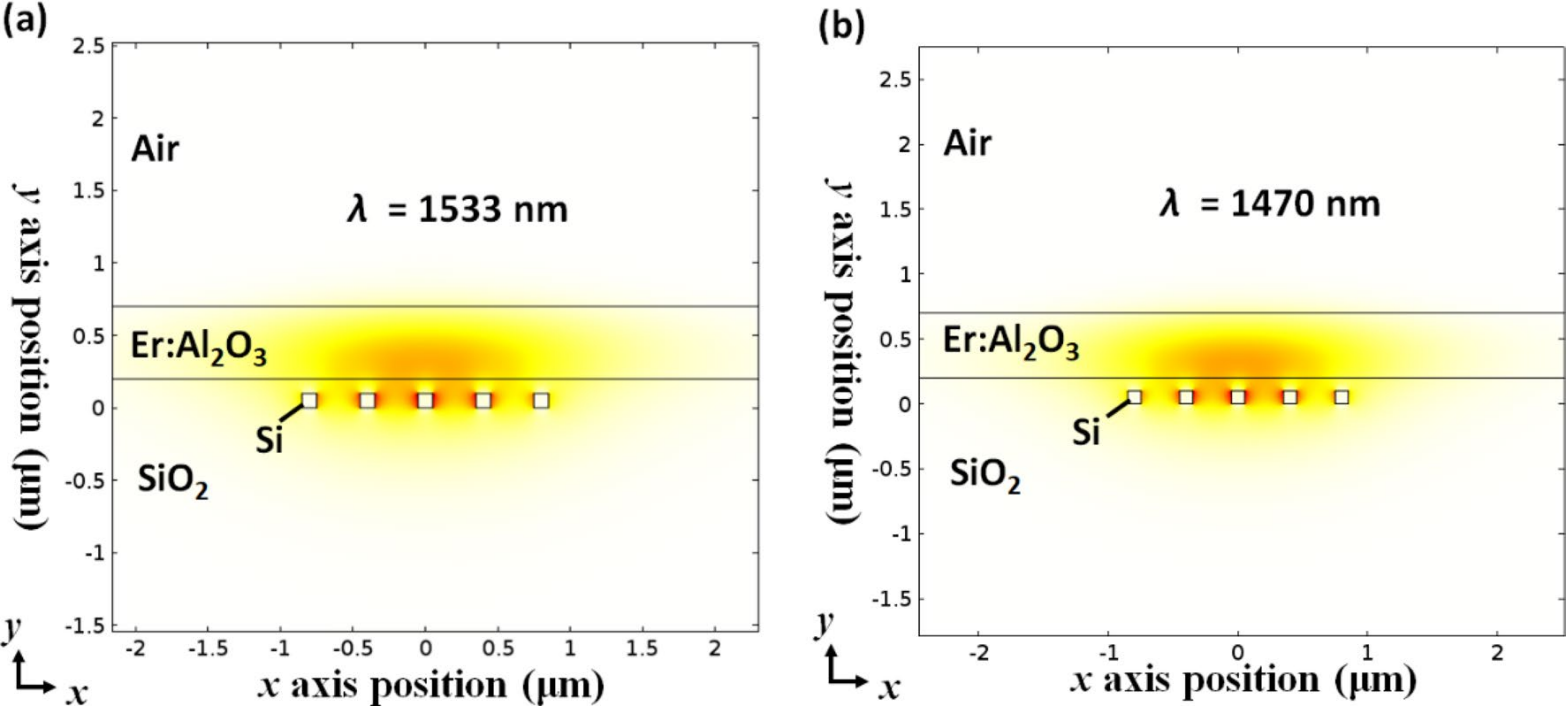
Mode analysis results obtained through *COMSOL Multiphysics* simulation under the following waveguide dimentsions: *h*_*Er*_ = 500 nm, *w*_*Si*_ = 100 nm, *h*_*Si*_ = 100 nm, *gap* = 300 nm, *gox* = 100 nm. (**a**) |E_x_|^2^ plot at the signal wavelength, *λ*_*S*_ = 1533 nm. (**b**) |E_x_|^2^ plot at the pump wavelength, *λ*_*P*_ = 1,470 nm.

In order to illustrate the mode profiles of the pump and signal beams propagating in typical DFB waveguide structures, we investigate mode analysis results under the following waveguide dimensions: *h*_*Er*_ = 500 nm, *w*_*Si*_ = 100 nm, *h*_*Si*_ = 100 nm, *gap* = 300 nm, *gox* = 100 nm in Fig. 2a, b at the pump and signal wavelengths, respectively. From these mode profiles, we can see that a large part of the mode electric field is distributed in the active material region for both the pump and signal fields. We estimated the intermodal pump/signal overlap factor at 99.88%. Additionally, we estimated that the dielectric energy confinement factor in silicon was less than 0.05. Besides, this shows the benefit of using such a configuration for strongly minimizing the effect of TPA occurring in silicon.

To determine the electric field distributions of the propagating modes in the active material, we estimated their confinement factors in the Er:Al_2_O_3_ active layer *Γ*_*a*_ according to:

1 $$ \Gamma_{a} = \frac{{\iint_{A} {\varepsilon \left| E \right|^{2} dxdy}}}{{\iint_{\infty } {\varepsilon \left| E \right|^{2} dxdy}}}, $$

where *A* stands for the active region and ∞ stands for the full waveguide cross-section. To rigorously calculate the confinement factor relating the modal gain to the bulk material gain in a high index contrast waveguide, the group index of the mode (*n*_*g*_) should also be considered^25^. The confinement factor should thus be recast as:

2 $$ \Gamma = \frac{{n_{g} \iint_{A} {\varepsilon \left| E \right|^{2} dxdy}}}{{n_{A} \iint_{\infty } {\varepsilon \left| E \right|^{2} dxdy}}}, $$

where *n*_*A*_ is the refractive index of active gain material. In the present case of a multi-segment Si waveguide, *n*_*g*_ is yet about 1.68, and *n*_*A*_ about 1.65, with thus a *n*_*g*_/*n*_*A*_ ratio of only 1.018. In the course of our investigations, this small correction was thus ignored.

**Figure 3:**
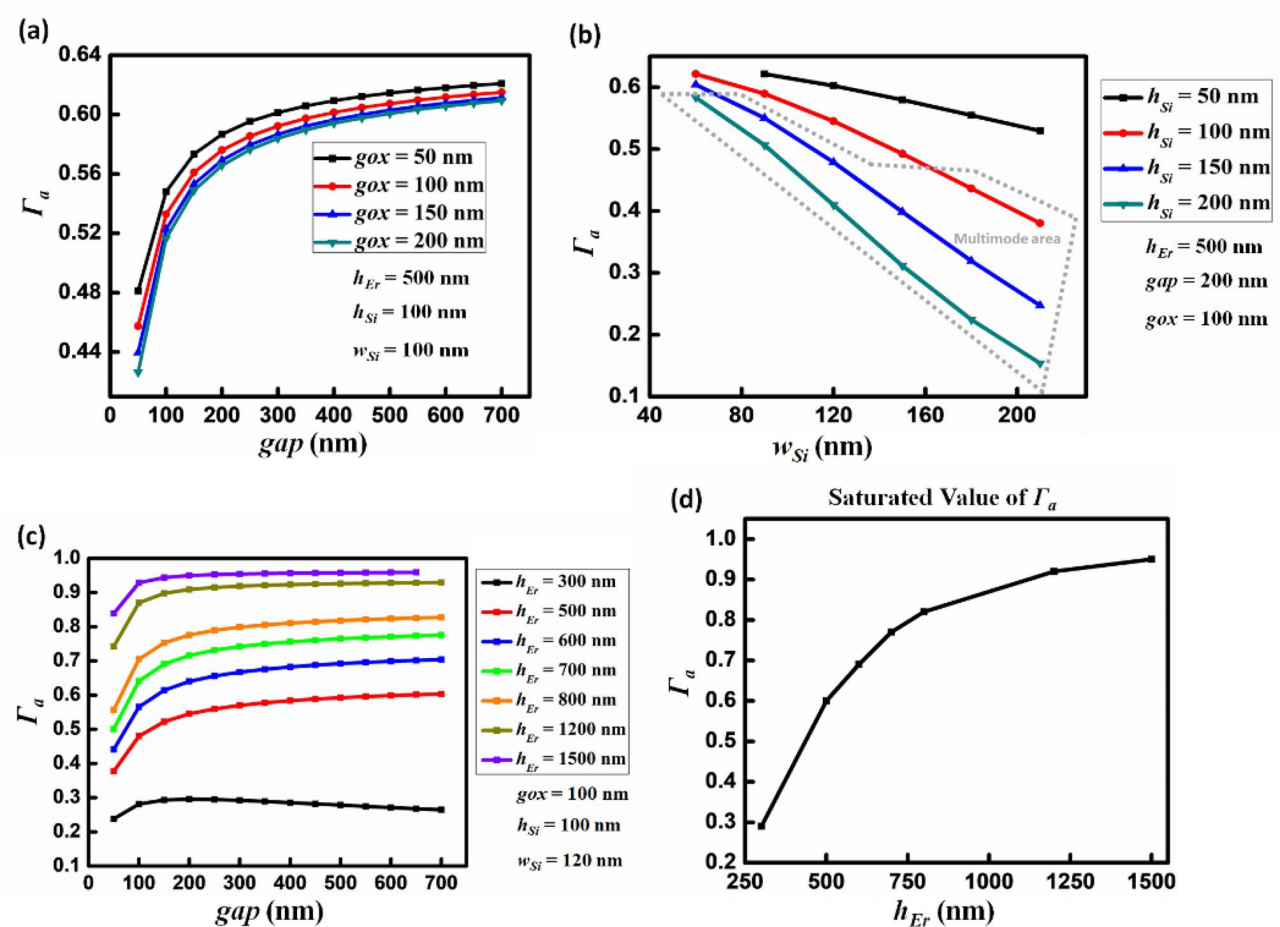
Exploring the influence of the waveguide parameters (see Fig. 1) on the dielectric energy confinement factor *Γ*_*a*_ in the active material: (**a**) *h*_*Er*_ = 500 nm, *h*_*Si*_ = 100 nm, *w*_*Si*_ = 100 nm, as a function of the *gap* and *gox* parameters. (**b**) *h*_*Er*_ = 500 nm, *gap* = 200 nm, *gox* = 100 nm, as a function of the *w*_*Si*_ and *h*_*Si*_ parameters*.* (**c**) *h*_*Er*_ = 500 nm, *h*_*Si*_ = 100 nm, *w*_*Si*_ = 120 nm, as a function of the *gap* and *h*_*Er*_ parameters. (**d**) Saturated value of *Γ*_*a*_ in different cases of *h*_*Er*_. A more in-depth discussion of the sensitivity of the results to the technological variability of the parameters is made in the final section, which provides a synthesis of the proposed laser configurations.

Our approach was to make the main parameters of the multi-segment waveguide sweep to study their influence on the value of *Γ*_*a*_, while keeping in mind the technological feasibility constraints, particularly those arising from the lithography and etching stages, and the sensitivity of the results to parameter variability. We started with a moderate situation with respect to the minimal feature sizes driven by lithography and etching constraints of the silicon rails: *w*_*Si*_ = 100 nm and *h*_*Si*_ = 100 nm, as well as *h*_*Er*_ = 500 nm as the initial choices. The inter-segment distance (*gap*) was then varied from 50 to 700 nm in 50 nm successive steps, while *gox* was varied from 50 to 200 nm in 50 nm successive steps. We thus obtained the mode solutions for all the parameter combinations. Figure 3a–c give a synthesis of the main results in terms of the confinement factor *Γ*_*a*_. From Fig. 3a, we can see that when *gap* increases before reaching 200 nm, *Γ*_*a*_ also increases, while when *gap* increases from 200 to 700 nm, the increment of *Γ*_*a*_ tends to saturate. Another conclusion derived from Fig. 3a is that the influence of *gox* on *Γ*_*a*_ is weak, especially when *gap* is larger than 200 nm. An inter-segment distance above 200 nm should thus be chosen, while the value of *gap* is not very sensitive on the confinement factor and can be selected flexibly according to fabrication constraints.

Moreover, in order to get enough information on the influence induced by the dimension of the silicon segments, we choose *h*_*Er*_ = 500 nm, *gap* = 200 nm, *gox* = 100 nm, and then swept *h*_*Si*_ and *w*_*Si*_ . We considered *h*_*Si*_ to vary from 50 to 200 nm and *w*_*Si*_ from 60 to 210 nm. The results are plotted in Fig. 3b. Figure 3b shows that when the silicon film thickness (*h*_*Si*_) is constant, *Γ*_*a*_ decreases when the silicon rails width (*w*_*Si*_) increases. Furthermore, we observe that when *h*_*Si*_ increases, the stronger the decrease of *Γ*_*a*_ is. When *w*_*Si*_ is constant, *Γ*_*a*_ is lowered when *h*_*Si*_ increases. Additionally, *w*_*Si*_ and *h*_*Si*_ can easily reach combinations such that a multimode operation occurs, as shown in the gray dotted area in Fig. 3b. Overall, *w*_*Si*_ and *h*_*Si*_ should thus be maintained at small enough values, which is not a drawback as large values of these two parameters tend to lower *Γ*_*a*_. Another point worth noting is that no result is obtained when *w*_*Si*_ = 60 nm, *h*_*Si*_ = 50 nm because no effective guided mode is found in this situation for which the silicon core part is too narrow to allow proper light confinement at the operating wavelength. From this analysis, we conclude that a good compromise between the waveguiding condition and minimal size constraints compatible with clean room fabrication tolerances was to set both the *w*_*Si*_ and *h*_*Si*_ parameters to ~ 100 nm.

In the above analysis, one parameter was never adjusted: the thickness of the active material (*h*_*Er*_). In fact, although *h*_*Er*_ has a very important influence on the results, our investigations led us to observe that this parameter roughly led to a translation of the optimum obtained by varying the other parameters. As a result, the parameter set can be optimized with the exception of *h*_*Er*_, and then *h*_*Er*_ can be adjusted as a last resort. We investigated the relation between *h*_*Er*_ and *Γ*_*a*_ according to the previous study by fixing the *h*_*Si*_ = 100 nm, *w*_*Si*_ = 120 nm and then swept the value of *gap* for seven different active layer thickness values: *h*_*Er*_ values: 300 nm, 500 nm, 600 nm, 700 nm, 800 nm, 1,200 nm and 1,500 nm, respectively. Figure 3c shows the related results. From Fig. 3c, it is obvious that the active layer thickness has a strong influence on the mode confinement factor in the active layer while *gap* is only a second order parameter, which thus proves its little sensitivity to the fabrication errors. If *h*_*Er*_ is constant and *gap* is less than 200 nm, *Γ*_*a*_ increases when *gap* increases. When *gap* is larger than 200 nm, *Γ*_*a*_ tends to saturate. However, the saturated value of *Γ*_*a*_ is strongly related to *h*_*Er*_: the larger *h*_*Er*_, the higher value of *Γ*_*a*_.

Combined with the previous results shown in Fig. 3a, b, we can clearly draw the following conclusion: *Γ*_*a*_ is dominated by *h*_*Er*_, the thickness of the Er:Al_2_O_3_-layer. Figure 3d shows the value level of *Γ*_*a*_ when it is saturated for different thickness values of the Er:Al_2_O_3_-layer, which will provide important guidance for laser design in the next step.

In summary, two conclusions can be drawn from the results reported in this section. Firstly, the mode confinement factor in the active material can be adjusted in a wide range and its value can reach up to 95%, which is a significant interest for achieving a strong laser effect. Secondly, the set of parameters leading to this confinement factor is only weakly sensitive to variations in the opto-geometric parameters of the multi-segment silicon waveguide, which induces a significant robustness of the investigated active structure to technological variations of the clean room fabrication processes.

### Threshold analysis of compact QWS-DFB laser

**Figure 4:**
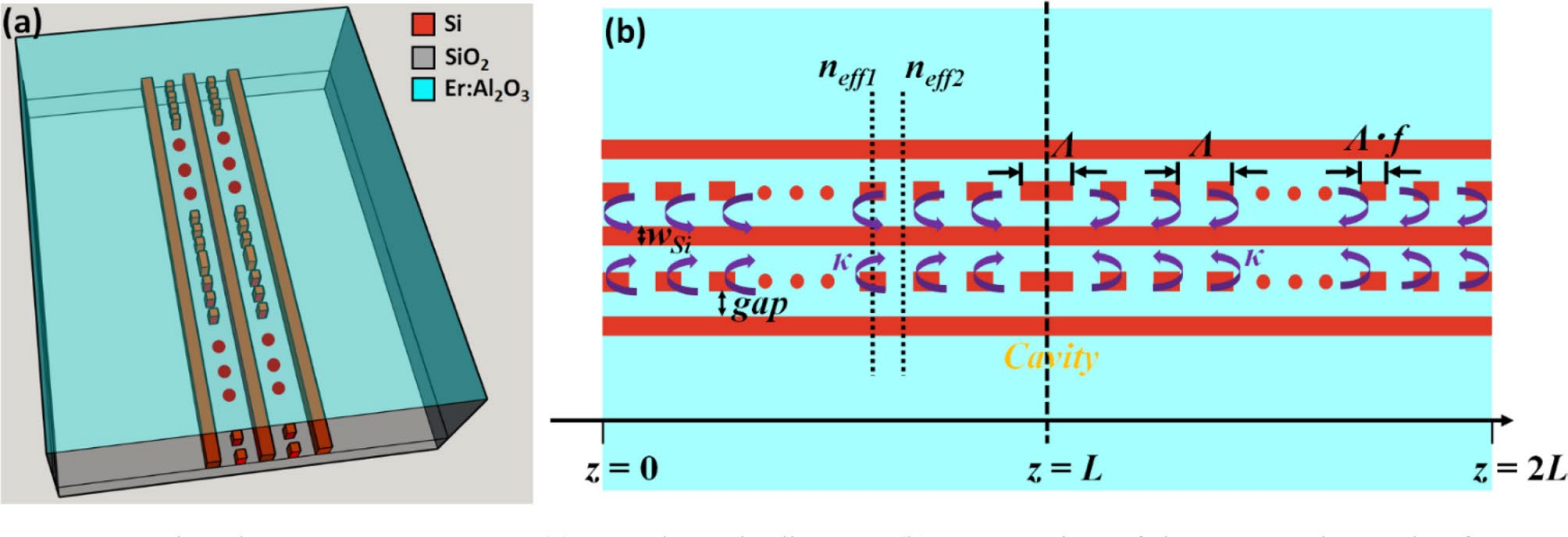
Designed QWS-DFB structure. (**a**) 3D schematic diagram. (**b**) 2D top view of the DFB cavity made of two identical DBR left and right mirrors and a quarter-wave intermediate section.

Based on the waveguide structure mentioned above, QWS DFB lasers can be flexibly designed as the considered composite waveguide contains several parallel rails and that patterning several of them with slits allows realizing Bragg mirrors with controlled and widely adjustable mirror strengths. A QWS-DFB laser structure is composed of two distributed Bragg reflectors (DBR), which are set back to back with a zero gap. The 3D schematic diagram of the designed QWS-DFB structure is shown in Fig. 4a,b shows its top view. We choose the two silicon segments which are closer to the center one to design distributed gratings. In this way, appreciable grating coupling coefficient (|*κ*|) can be obtained without a huge effect on the target waveguide mode. The length of the distributed Bragg mirrors in each side is *L*. The grating period is *Λ*, and the filling factor *f* (defined as the ratio of single silicon grating length to the grating period) is 0.5, which provides the highest coupling coefficient^26^.

With this structure defined, the critical point to be evaluated is to study the conditions that can lead to reach the laser threshold condition under the cumulative effects of the losses arising from the active medium, the losses arising from the DBR mirrors, and the modal gain arising from the active medium under optical pumping at 1470 nm wavelength. Hereafter, *g*_*th*_ stands for the material gain at lasing threshold and *α* is the active material absorption loss level, *g*_*mat*_ = (*g*_*th *_− *α*) thus being the net material gain under pumping. *α*_*m*_ is the mirror loss level and *α*_*s*_ is the waveguide scattering loss coefficient. The lasing condition to be satisfied is then:

3 $$ \Gamma_{a} (g_{th} - \alpha ) = \alpha_{m} + \alpha_{s} . $$

Here, we first provide an analysis for the calculation of *α*_*m*_ (mirror losses) through the coupled mode theory (CMT) according to Ref.^27^. First, we calculate the reflectivity of a single distributed Bragg mirror according to the CMT:

4 $$ \left\{ {\begin{array}{*{20}l} {r = \frac{{ - \kappa^{*} \sinh (sL)}}{\Delta \beta \sinh (sL) + is\cosh (sL)}} \hfill \\ {\Delta \beta = \beta - \beta_{0} = \beta ^{\prime} - i\Gamma_{a} \frac{g}{2} - \beta_{0} } \hfill \\ {s = \sqrt {\left| \kappa \right|^{2} - \Delta \beta^{2} } } \hfill \\ \end{array} } \right. $$

where ***κ*** is the grating coupling coefficient, *L* is the length of DBR, *g* is the material gain and *β* is the mode propagation vector. Assuming zero internal loss cavity in the first step, the roundtrip condition for lasing in such cavity is simply:

5 $$ r^{2} = \left[ {\frac{{ - \kappa^{*} \sinh (sL)}}{\Delta \beta \sinh (sL) + is\cosh (sL)}} \right]^{2} = 1. $$

In order to estimate the threshold condition for the laser effect, it is sufficient to consider Eq. (5) for the fundamental mode of the QWS-DFB laser for which the wavelength *β*' − *β* = 0. Then we can simplify Eqs. (4) and (5) into Eq. (6):

6 $$ \left[ {\frac{{ - \kappa^{*} \sinh [\sqrt {(\left| \kappa \right|L)^{2} + \left( {\Gamma_{a} \frac{g}{2}L} \right)^{2} } ]}}{{ - i\Gamma_{a} \frac{g}{2}\sinh [\sqrt {(\left| \kappa \right|L)^{2} + \left( {\Gamma_{a} \frac{g}{2}L} \right)^{2} } ] + i\sqrt {\left| \kappa \right|^{2} + \left( {\Gamma_{a} \frac{g}{2}} \right)^{2} } \cosh [\sqrt {(\left| \kappa \right|L)^{2} + \left( {\Gamma_{a} \frac{g}{2}L} \right)^{2} } ]}}} \right]^{2} = 1 $$

**Figure 5:**
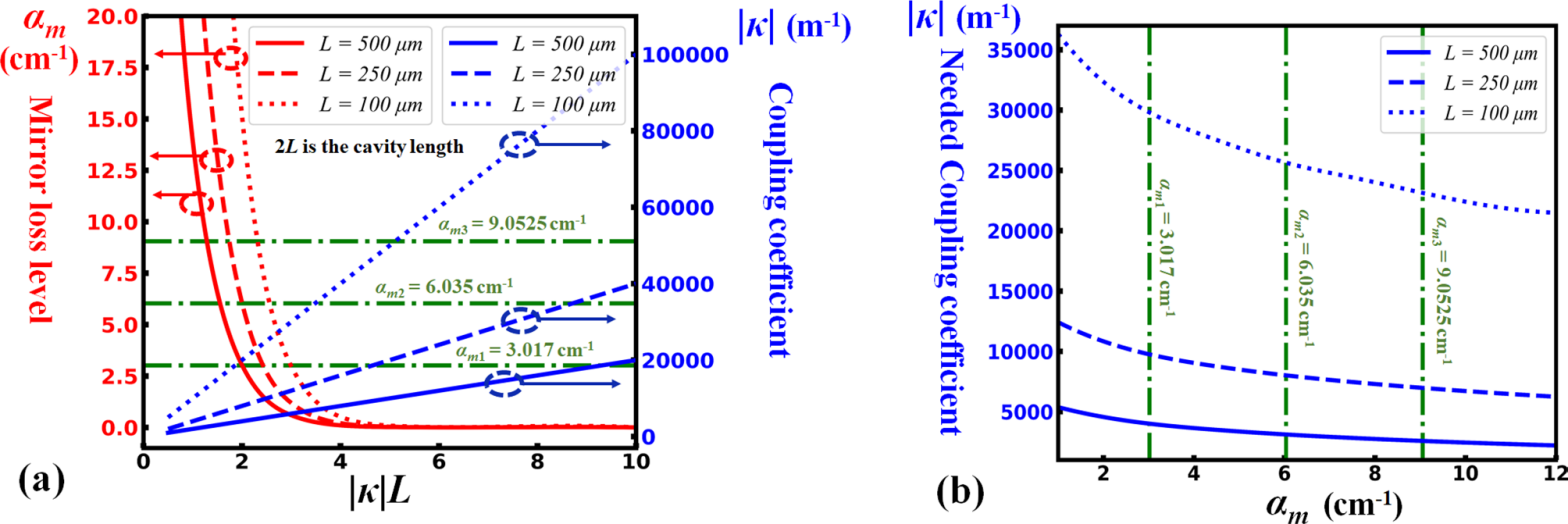
(**a**) Calculation results for *α*_*m*_ (mirror loss level) in different situations of |*κ*|*L* as well as the corresponding |*κ*| values at each *L* (DBR mirror length) value. *α*_*m*1_, *α*_*m*2_ and *α*_*m*3_ correspond to the laser conditions (*α*_*m*_ = *Γ*_*a*_*g*_*mat*_) when the confinement factor (*Γ*_*a*_) is 0.25, 0.5, and 0.75, respectively. (**b**) The relationship between *α*_*m*_ and the needed |*κ*| values to satisfy the lasing condition in different situations of the DBR mirror length (*L*).

Given the coupling coefficient |*κ*| and the length *L*, the coupling constant |*κ*|*L* can then be calculated. The corresponding value of *Γ*_*a*_ × *g* can be obtained through solving Eq. (6). Finally, the mirror loss *α*_*m*_ thus equals to *Γ*_*a*_ × *g*. Figure 5a presents a synthesis of the obtained results in different conditions, as well as the corresponding values of |*κ*| and *α*_*m*_ values when *L* is 500 μm, 250 μm and 100 μm, respectively. It is noteworthy that these dimensions are significantly shorter compared to the typical dimensions of the previously reported DFB lasers^16–22^.

According to Ref.^23^, a net material gain *g*_*mat*_ = 12.07 cm^−1^ was monitored under an optical pumping power of few mW, and we therefore consider it here as a realistic reference level of the net gain conditions that can be obtained. As can be seen in Fig. 5a, the cavity Bragg mirror losses *α*_*m*_ (expressed in cm^−1^ for comparison with propagation losses) can be severely minimized when |*κ*|*L* >> 2. At the same time, this condition is somewhat contrary to the realization of compact lasers since it is predictable that the coupling coefficient of the mirrors |*κ*| is intrinsically limited by the geometry of the periodically textured silicon waveguides. This is the reason why, operating around or below |*κ*|*L* = 2 deserves attention. In this region, the waveguide scattering losses of standard silicon waveguides at near infrared wavelengths (*α*_*s*_ < 0.25 cm^−1^) can be ignored compared with the mirror losses (*α*_*m*_). Consequently, the lasing condition then becomes: *Γ*_*a*_* g*_*mat*_* ≈ α*_*m*_ This simple equation shows that the maximum tolerance level of the cavity mirror losses *α*_*m*_ depends essentially on the net modal gain that can be achieved in the active medium, and thus on the mode confinement factor *Γ*_*a*_ in the active region. The added three horizontal green lines in Fig. 5a correspond to the three mirror losses levels: *α*_*m*1_ = 0.25*g*_*mat*_ (~ 3.0 cm^−1^), *α*_*m*2_ = 0.50*g*_*mat*_ (~ 6.0 cm^−1^), and *α*_*m*3_ = 0.75*g*_*mat*_ (~ 9.0 cm^−1^), respectively. As such, the required values of |*κ*|*L* can be derived for each mirror loss level and cavity length of 2*L*. The needed coupling coefficient values at different values of *α*_*m*_ and *L* are quantitatively shown in Fig. 5b. The main information resulting from these estimates is that an effective feedback coefficient |*κ*|*L* of ~ 2 and Bragg mirror coupling coefficients |*κ*| ranging between 5,000 and 30,000 m^−1^ are sufficient to limit the losses of the Bragg mirrors of a distributed active cavity below the level of the typical modal gain level that can be achieved in erbium-doped hybrid multi-rail silicon waveguides optically pumped at ~ 1.48 µm.

### Seeking the DBR strength to reach the lasing condition with a high ***Γ***_***a***_ in sub-mm waveguide structure

**Figure 6:**
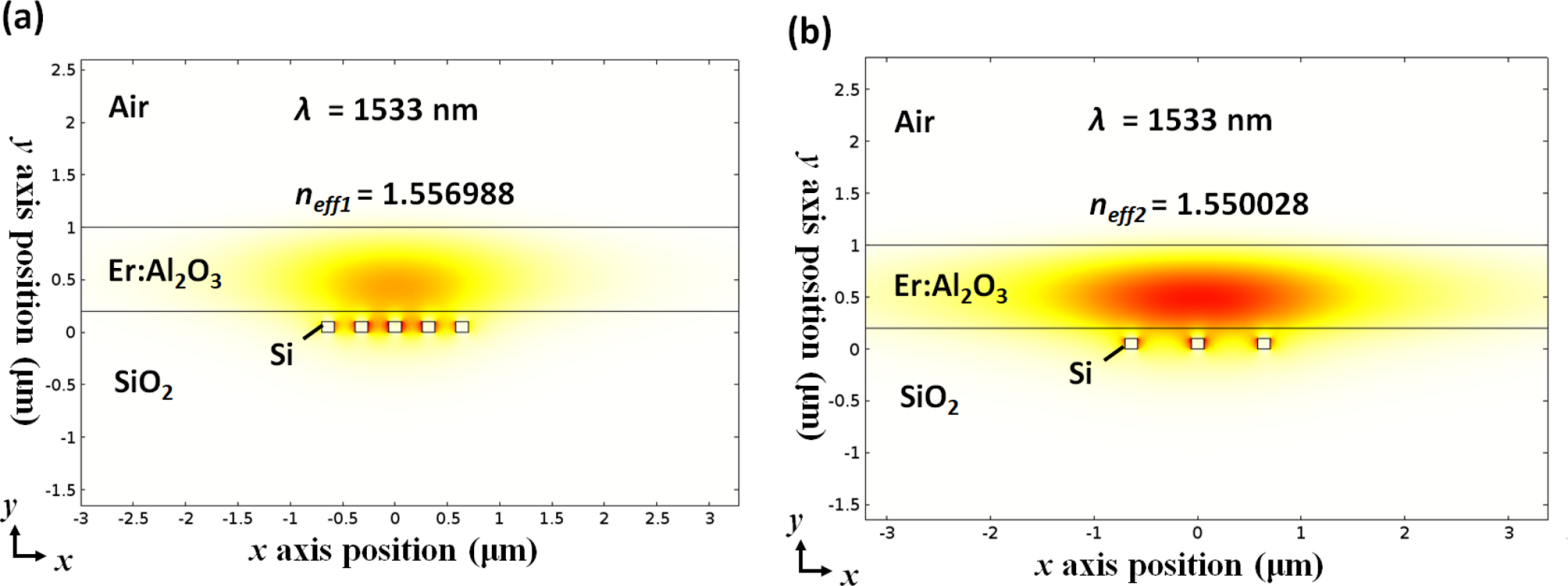
Mode analysis results for signal wavelength when the parameters of waveguide structure are as follows: *h*_*Er*_ = 800 nm, *w*_*Si*_ = 120 nm, *h*_*Si*_ = 100 nm, *gap* = 200 nm, *gox* = 100 nm. (**a**) |E_x_|^2^ plot in the un-corrugated multi-segments waveguide. (**b**) |E_x_|^2^ plot in the corrugated multi-segments waveguide.

Bragg mirrors have been widely considered in several works for the realization of passive components such as filters or for the design of laser cavities^28,29^. We simply address the potential realization of Bragg mirrors based on the particular geometry of the multi-rail silicon waveguide geometry described in section "Results". Segmented waveguide Bragg mirror configurations (see Fig. 4) are considered and the main equations that are used are based on the coupled mode theory. |*κ*| can be interpreted as the amount of light reflection per unit length^30^. By considering stepwise effective index variations, waveguide sections with and without a grating corrugation, respectively, can be considered for the estimation of |*κ*|. As previously indicated, a corrugation of the first two side rails with respect to the central rail is considered for the realization of the distributed optical feedback mechanism. We label the two effective indices as *n*_*eff1*_ and *n*_*eff2*_ for the regions with and without grating corrugation, respectively, as shown in Fig. 4b. The reflection at each interface can be written as (*n*_*eff1  *_− *n *_*eff2*_)/2*n*_*eff*_ according to the Fresnel equations, where *n*_*eff*_ is the effective index of Bragg wavelength (*λ*_*B*_) in the waveguide. Each grating period contributes to two reflections. Therefore, the coupling coefficient |*κ*| can be estimated as:

7 $$ \left| \kappa \right| = 2\frac{{\left| {n_{eff1} - n_{eff2} } \right|}}{{2n_{eff} }}\frac{1}{\Lambda } = \frac{{2\left| {n_{eff1} - n_{eff2} } \right|}}{{\lambda_{B} }}, $$

where *λ*_*B*_ = 2*n*_*eff*_* Λ*. In order to target a high *Γ*_*a*_ situation, we firstly choose *h*_*Er*_ = 800 nm to explore the possible achievable values for the Bragg mirror coupling coefficient |*κ*|. Combined with the analysis presented in section "Conclusion", the multi-rail silicon waveguide parameters are selected as follows: *w*_*Si*_ = 120 nm, *h*_*Si*_ = 100 nm, *gox* = 100 nm and *gap* = 200 nm. In order to qualitatively guide our analysis, we plot in Fig. 6a,b the obtained modes profiles in the two un-corrugated (*n*_*eff1*_ = 1.556988) and corrugated (*n*_*eff2*_ = 1.550028) waveguide sections at *λ*_*B*_ = 1533 nm as the signal wavelength, from which we derive |*κ*|= 9,080 m^−1^ through Eq. (7). Additionally, a confinement factor *Γ*_*a*_ = 0.776 was also obtained. As such, we see that |*κ*|*L* values of 4.54, 2.27, and 0.908, respectively can be readily obtained for *L* = 500 μm, *L* = 250 μm, and *L* = 100 μm, corresponding to *α*_*m*_ values of 0.0372 cm^−1^, 3.9536 cm^−1^, 80.14 cm^−1^. By comparing the value of *α*_*m*_ with *Γ*_*a*_*g*_*mat*_, we can figure out the lasing feasibility for *L* = 500 μm and *L* = 250 μm but not for *L* = 100 μm. It can thus be seen from the outset that when *L* = 500 μm and *L* = 250 μm, the coupling force values required to reach the laser threshold in the corresponding structures using a periodically corrugated multi-segment silicon/active ALD coating waveguide in the proposed configuration is quite easily achievable, while no specific optimization has been carried out at this stage. Obviously, more optimization is yet needed to fix the limits of the best length/gain compromise.

**Figure 7:**
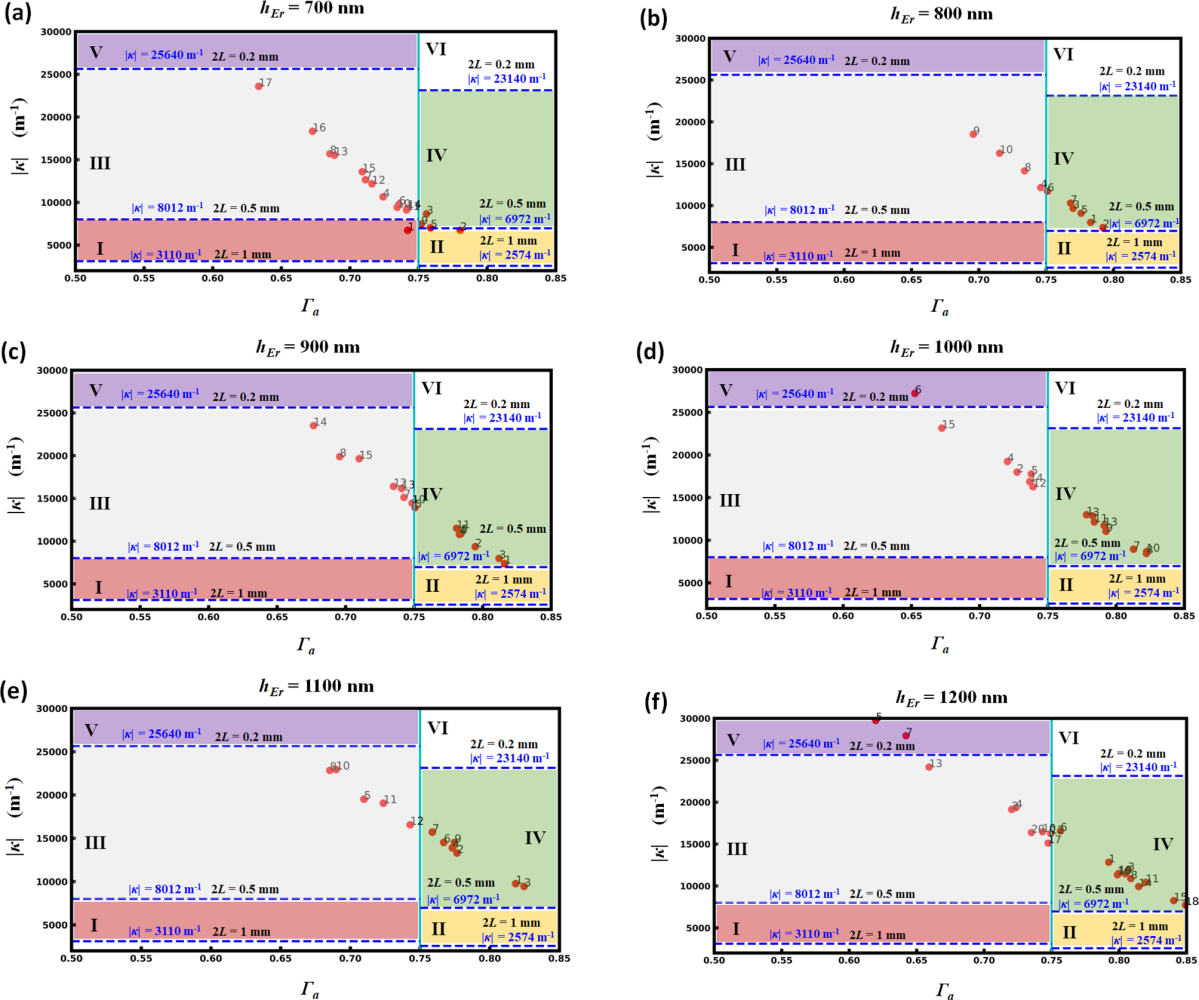
Exploration of the relationship between |*κ*| and *Γ*_*a*_ in different situations. (**a**) *h*_*Er*_ = 700 nm. (**b**) *h*_*Er*_ = 800 nm. (**c**) *h*_*Er*_ = 900 nm. (**d**) *h*_*Er*_ = 1,000 nm. (**e**) *h*_*Er*_ = 1,100 nm. (**f**) *h*_*Er*_ = 1,200 nm. For each plot of **a**–**f**, the number labels stand for different combinations of the waveguide parameters (details are given in the “Supplementary information” section).

As it is known, the relationship between |*κ*| and *Γ*_*a*_ stems on a trade-off. Indeed, a large confinement of light in the low index active material intrinsically means that the effect of the Bragg corrugation performed in the silicon rails is less efficient (all other parameters being constant), i.e. is weaker. We have thus explored the values of *Γ*_*a*_ and |*κ*| in different situations. A higher *Γ*_*a*_ is primary for avoiding the TPA effect, then we have selected six different values of the active material thickness (*h*_*Er*_): 700 nm, 800 nm, 900 nm, 1,000 nm, 1100 nm, and 1,200 nm, respectively. For each *h*_*Er*_ value, many different parameters combinations have been scanned and studied. The cases situated around the lasing criterion have been readily retained. The related results in terms of *Γ*_*a*_ and |*κ*| parameters are plotted in Fig. 7a–f, according to the values of *h*_*Er*_, respectively.

Figure 7a shows the obtained results for *h*_*Er*_ = 700 nm: seventeen scatter points are labeled corresponding to seventeen combinations of parameters and the detailed information of these parameters are in the “Supplementary information” section. In Fig. 7a, the starting point of the *x* coordinate axis is *Γ*_*a*_ = 0.50, which is the lowest limit that we set. The vertical cyan solid line stands for the symbol of *Γ*_*a*_ = 0.75. According to the results in Fig. 5b, we can see that when *Γ*_*a*_ = 0.50, the DFB laser length 2*L* can reach 1 mm if |*κ*| ≥ 3,110 m^−1^, 0.5 mm if |*κ*| ≥ 8,012 m^−1^ and 0.2 mm if |*κ*| ≥ 25,640 m^−1^. When *Γ*_*a*_ = 0.75, the DFB laser length 2*L* can reach 1 mm if |*κ*| ≥ 2,574 m^−1^, 0.5 mm if |*κ*| ≥ 6,972 m^−1^ and 0.2 mm if |*κ*| ≥ 23,140 m^−1^. All the corresponding |*κ*| values are shown in Fig. 7a as blue dashed lines. Based on the lasing condition that both |*κ*| and *Γ*_*a*_ values can meet these requirements simultaneously, we can divide the Fig. 7a into six regions (I–VI). Region I stands that a 1 mm long length DFB laser can be achieved with a confinement factor lager than 0.50 meanwhile smaller than 0.75 and all other regions are labeled accordingly. Figure 7b–f own the same properties and organization as Fig. 7a, and they correspond to the cases of *h*_*Er*_ = 800 nm, *h*_*Er*_ = 900 nm, *h*_*Er*_ = 1,000 nm, *h*_*Er*_ = 1,100 nm, *h*_*Er*_ = 1,200 nm, respectively. By observing Fig. 7a–f, we can see that there are a lot of points located in the regions III and IV in each case of *h*_*Er*_ value, showing the straightforward feasibility of achieving 0.5 mm long DFB lasers. Additionally, we can find that there is one point (number 6) located in region V in Fig. 7d and two points (number 5, 7) located in region V in Fig. 7f. However, no point was found yet in region VI.

Simultaneously, the gathered results provide guidance with respect to the fabrication tolerances of the Bragg composite waveguide. As the thickness of the active Er:Al_2_O_3_ layer (*h*_*Er*_) can be controlled accurately (< 10 nm) through the ALD process, its influence on the gain threshold condition is weak in the range of the layer necessary thicknesses (see Fig. 3). Meanwhile, for each value of *h*_*Er*_, it appears that many close combinations of other parameters can meet the requirements to achieve lasing in sub-mm long footprints. For example, in Fig. 7d, making an analysis for the parameters of points labeled as number 8–14, conclusions can be obtained as follows: (1) when *gap* = 300 nm, *gox* = 100 nm, *h*_*Si*_ = 100 nm, the values of *w*_*Si*_ ranging from 160 to 200 nm are qualified; (2) when *gap* = 300 nm, *gox* = 100 nm, *w*_*Si*_ = 180 nm, the values of *h*_*Si*_ ranging from 80 to 120 nm are also qualified; (3) in most of cases, *gap* = 200 nm and *gap* = 300 nm can both be suitable. These conclusions thus provide confidence in the very acceptable fabrication geometrical tolerances of the proposed scheme with respect to deep-UV or e-beam lithography and etching standard silicon processes.

Here, based on the DFB laser strategy, we present an additional analysis of its lasing output slope efficiency $SE=\Delta {P}_{s}/\Delta {P}_{p}$, with *P*_*s*_ and *P*_*p*_ the signal and pump powers, respectively. *SE* is in fact the outcome of the laser external quantum efficiency (*η*_*e*_) and the ratio *λ*_*pump*_/*λ*_*signal*_ of the two involved signal wavelengths: *SE* = (*λ*_*pump*_/*λ*_*signal*_) × *η*_*e*_. The external quantum efficiency itself derives from the material internal efficiency (*η*_*i*_) and our ability to extract the emitted photons (*η*_*extract*_):

8 $$ \eta_{e} = \eta_{i} \times \eta_{extract} $$

The internal quantum efficiency is the ratio of radiative rate to total recombination rate, which in our case, is the ratio of the radiative rate (1/*τ*_*r*_) to the spontaneous emission rate of between the ^4^*I*_13/2_ first excited state (1/*τ*_*21*_) in the Er system:

9 $$ \eta_{i} = \frac{{1/\tau_{r} }}{{1/\tau_{21} }}. $$

The value for the radiative lifetime of Er-ions in Al_2_O_3_ is ~ 10.2 ms^31^ and the lifetime of state ^4^*I*_13/2_ is 2.05 ms in our case^23^, which means *τ*_*r*_ = 1/10.22 ms^−1^ and *τ*_*21*_ = 1/2.05 ms^−1^. Consequently, the internal quantum efficiency (*η*_*i*_) can be estimated to 20.06%. As for the extraction quantum efficiency, it can be derived from the laser cavity structure characteristics^32^ from Eq. (10):

10 $$ \eta_{extract} = \frac{1}{{1 - \alpha_{i} \times 2L/{\ln}\left( R \right)}} $$

where *α*_*i*_ is the intrinsic loss, 2*L* is the cavity total length and *R* is the reflectivity for signal wavelength. *R* can be derived by Eq. (11)^30^ as below:

11 $$ R = \tanh^{2} (\kappa L) $$

If we estimate the upper limit for the intrinsic material loss at the laser threshold by assuming steady-state threshold population inversion where half of the active ions remain in their ground state under pumping, then *α*_*i*_ ~ 4.03 cm^−1^ can be derived from Ref.^23^. Finally, the slope efficiency values of the DFB cavity silicon Erbium laser can be estimated with different laser lengths and corresponding necessary |*κ*| values. The results are summarized in Table 2.

**Table 2:** Estimated values of slope efficiency.

*2L* (cavity total length)	*Γ*_*a*_	*|κ|* (m^−1^)	*R*	*SE* (slope efficiency) (%)
1 mm	0.50	3,110	0.8365	5.91
1 mm	0.75	2,574	0.7367	8.30
500 µm	0.50	8,012	0.9298	5.10
500 µm	0.75	6,972	0.8847	7.27
200 µm	0.50	25,640	0.9766	4.37

Overall, we can conclude that less than 1 mm and even 0.5 mm, 0.2 mm length erbium-doped material hybrid integrated on-chip DFB lasers can be realized with several sets of Bragg mirror parameters with a fairly robust behavior with respect to fabrication errors of the silicon Bragg multi-segment composite structures. Their lasing output slope efficiency has been analyzed briefly and the value of *SE* is estimated as 4.37–8.30%. Noteworthy is that a reasonable margin design space should be considered for the practical fabrication of structures, both in term of confinement factor (*Γ*_*a*_) and Bragg mirror coupling coefficient (|*κ*|).

## Conclusion

In summary, we investigate the design of DFB lasers relying on composite silicon multiple-rail waveguides coated with a highly doped Er:Al_2_O_3_ layer grown by the ALD technique and optically pumped at 1470 nm wavelength. The waveguide mode properties are investigated for exploring the influence of the structure’s opto-geometrical parameters on the mode confinement factor in the active layer and on the Bragg mirrors’ strength. This analysis reveals that the lasing threshold calculated through the coupled mode theory by considering realistic experimentally reported material gain levels at 1533 nm wavelength can be reached for sub-mm active structures, even 0.5 mm and 0.2 mm length footprint size. It appears in fact that the design compromises leave a relatively large part to the variability of the opto-geometric parameters. As a result, a wide range of parameters are available for the realization of lasers directly integrated into silicon-on-insulator waveguides. All the results show the very high potential of oxides doped with erbium and deposited at low temperature by ALD for the realization of integrated lasers pumped at 1,470 nm by continuous sources of a few mW power^23^. This opens up interesting prospects for integrating and combining these sources to create optical links or more complex on-chip functions, and brings a contribution to the problem of sources and amplifiers for the silicon photonics platform.

## Methods

### Numerical simulation

The mode analysis results, including the confinement factors, were obtained through a finite element mode solver, *COMSOL Multiphysics*. The lasing threshold equations were solved with *MATLAB*.

## Supplementary information

Supplementary file1 (DOCX 166 kb)
