# Supplementary material for: Potential for sub-mm long erbium-doped composite silicon waveguide DFB lasers
Source: Sci Rep. 2020 Jul 2;10:10878. doi: 10.1038/s41598-020-67722-y (PMC7331813; doi:10.1038/s41598-020-67722-y)

Potential for sub-mm long erbium-doped composite silicon waveguide DFB lasers

Zhengrui Tu,^1^ Jianhao Zhang,^1^ John Rönn,^2^ Carlos Alonso-Ramos,^1^ Xavier Leroux,^1^ Laurent Vivien,^1^ Zhipei Sun,^2,3^ and Éric Cassan^1,*^

^1^Centre de Nanosciences et de Nanotechnologies (C2N), Université Paris Saclay, Université Paris Sud, CNRS, 91120 Palaiseau, France

^2^Department of Electronics and Nanoengineering, Aalto University, Tietotie 3, FI-00076 Espoo, Finland

^3^QTF Centre of Excellence, Department of Applied Physics, Aalto University, FI-00076 Aalto, Finland

* [eric.cassan@universite-paris-saclay.fr](mailto:eric.cassan@universite-paris-saclay.fr)

(Please see the next two pages)

We provide here the detailed parameters of the composite silicon Al_2_O_3_:Er_2_O_3_ waveguide distributed Bragg mirrors corresponding to the configurations reported in Fig. 7 of the article text.


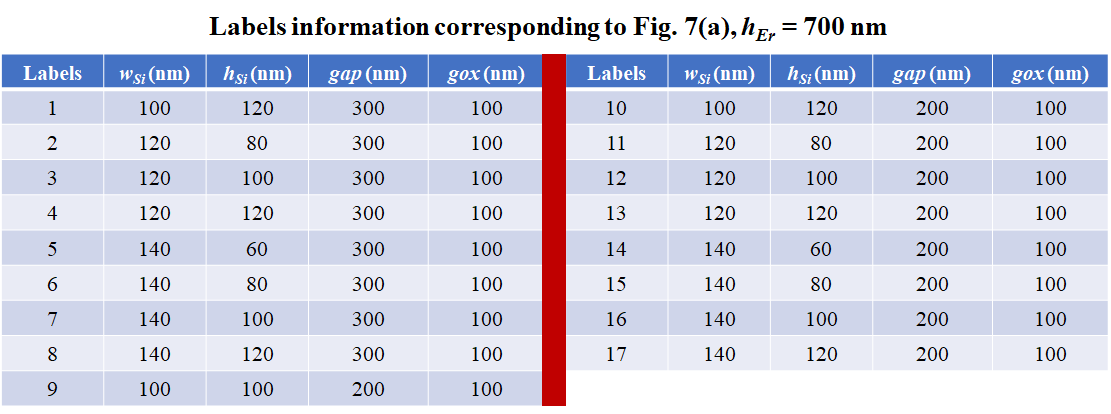


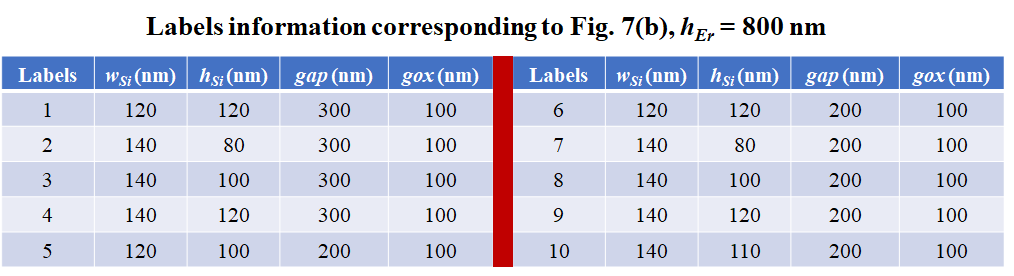


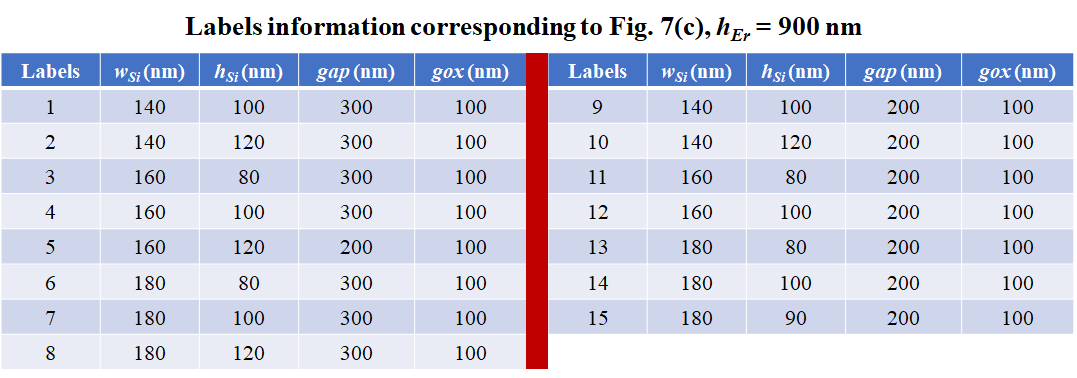


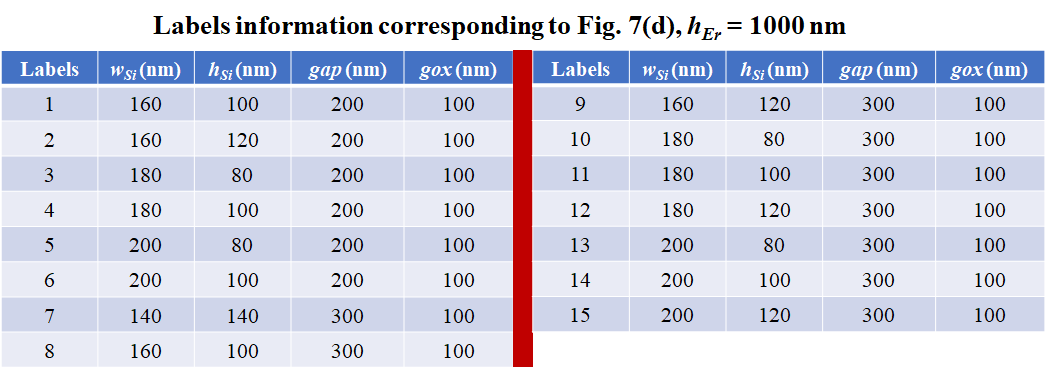


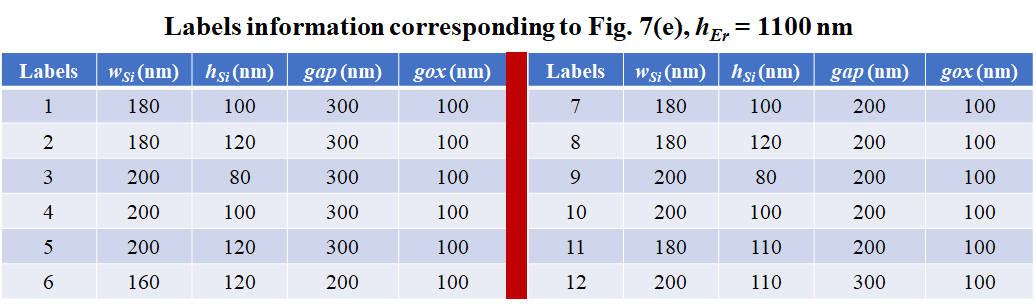


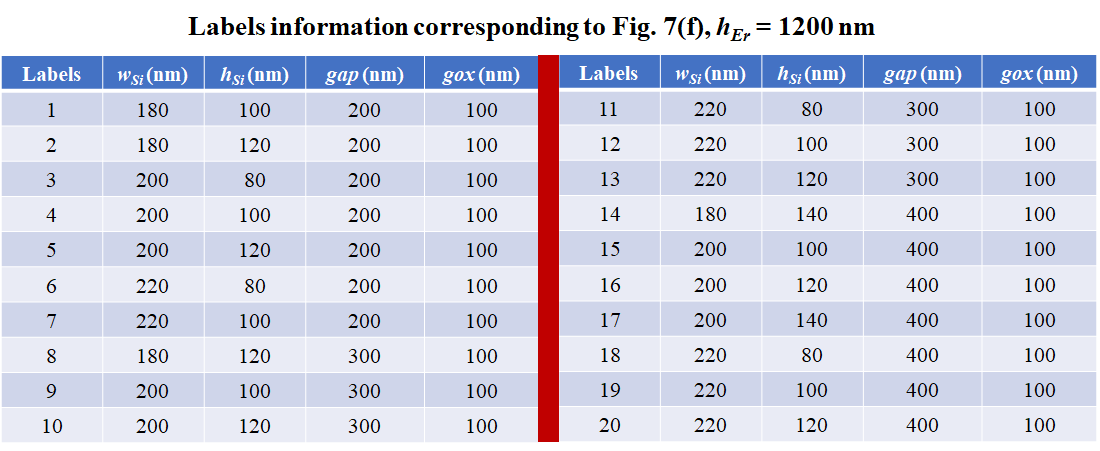

Supplement: Supplementary file 1 — Supplementary file1 (DOCX 166 kb) [file 41598_2020_67722_MOESM1_ESM.docx]
